# Contraceptive use and discontinuation among women aged 15–24 years in Kenya

**DOI:** 10.3389/frph.2023.1192193

**Published:** 2023-11-15

**Authors:** Wambui Kungu

**Affiliations:** Research and Centre of Excellence, National Council for Population and Development, Nairobi, Kenya

**Keywords:** contraceptive use, discontinuation, women age 15−24, Kenya, choices and challenges tool

## Abstract

**Introduction:**

The 15–24-year-old age group of young women make up about 15% of the population of 47 million Kenyans which comes to 7 million. Addressing the reproductive health goals of this cohort is thus a policy priority because of the high potential they pose for unintended pregnancy through incorrect and intermittent use of contraception.

**Objective:**

The study sought to present evidence on contraceptive use among women aged 15–24 in Kenya between 2012 and 2014 using Kenya Demographic and Health Survey (KDHS) 2014 Contraceptive calendar data and make recommendations on enhancing the correct and consistent use of contraception.

**Methodology:**

The data used was obtained from the Choices and Challenges tool developed by Population Reference Bureau (PRB) and visualized innovatively using Sankey Diagrams that show contraceptive use/non-use, continuation, switching/discontinuation, and pregnancy.

**Results:**

The use of contraceptives went up by about 30% during the study period while the use of modern methods went up by 83%. The uptake of Long-Acting Reversible Contraception (LARC) went up by 87% while that of Short Acting Methods (SAM) went up by 70% but the progress was clouded by discontinuation rates of 35% with side effects being the leading reason for the abandonment of contraception.

**Conclusion:**

For Kenya to achieve transformative results in ending the unmet need for contraception and preventable maternal deaths, it is critical to sustaining the current gains in contraceptive prevalence rate (CPR) by promoting the retention of youth users and encouraging new users.

## Introduction

Trends in contraceptive use in Kenya between 2003 and 2014 have revealed a huge increase in contraceptive uptake among women aged 15–24 years with the use of long-term methods having increased fourfold from 2008/09 to 2014. The uptake of Implants rose rapidly and more than doubled to reach 21% in 2014 from 10% in 2008/09 (1). The 15–24-year old age group of young women made up about 15% of the population of 47 million Kenyans in 2019 which comes to 7 million (2). The group is therefore critical to economic development in view of the aspiration of achieving the Kenya Vision 2030 target of 10% economic growth per annum by 2030. Addressing the reproductive health goals of these adolescents and young women aged 15–24 years is therefore a policy priority because of their high risk of unintended pregnancy. The use of contraception is a game-changer in preventing unintended pregnancies, which pose risks of poor pregnancy outcomes, especially for teenage girls and it enhances the overall quality of life and status for women (3).

Among these young women, 62% of those using modern methods of contraception indicated that they do not wish to become pregnant (1). However, the cohort of women had the highest rates of discontinuation while still in need of contraception among all age groups with age and method of contraception being identified as the two significant determinants of discontinuation (4). Studies have found that in low and middle income countries, up to 20% of unmet need results from discontinuation due to side effects while about 30% of unintended births are attributed to method failure (5). However, discontinuation may not necessarily be negative and may be driven by the desire to get pregnant or switch to a more effective method. Youth who are not married may also discontinue because their sexual activity maybe sporadic but unmarried adolescents have the highest rates of discontinuation.

The Constitution of Kenya, 2010, the Health Act of 2017, and Sustainable Development Goal (SDG), indicator 3.7 have enshrined the right of every woman to safe, effective, affordable, and acceptable contraception services. Also in place is the Family Planning Costed Implementation Plan (FPCIP 2021–2024) whose goal is to reduce unmet need from 18% to 9% by 2024 and the National Adolescent Sexual and Reproductive Health policy (ASRHP), 2015 to guide on enhancing the status of sexual and reproductive health for adolescents (6). A Global Consensus Statement on expanding contraceptive options for adolescents and youth was issued by FP2020 (7) while Kenya's FP2030's focus is on enhanced access to acceptable, affordable, and equitable quality contraceptive services to ensure attainment of zero unmet need. FP2030 Commitment 2 aims to reduce unmet need from 14% to 10% by 2030 while Commitment 5 seeks to reduce adolescent pregnancy from 14% to 10% by 2025. Despite the many interventions to improve youth reproductive health, a lot of barriers still persist.

The International Conference on Population and Development (ICPD) of 1994 brought the issue of enhanced access to reproductive health (RH) to the forefront of the process of development. A key aspect of RH is contraception which refers to the preparation, knowledge, and methods that assist individuals and couples to plan and attain their desired family size and determine the spacing of pregnancy. Ending the unmet need for contraception and preventable maternal deaths are transformative result areas for the Government of Kenya in implementing the country's commitments of ICPD25 and in achieving Universal Health Coverage by 2030 (8).

Young women aged 15–24 years have multiple reproductive health needs which need a comprehensive, multi-sectoral approach to address them. Youth-friendly sexual and reproductive health services that can deal with the double threat of unintended pregnancy and HIV/AIDS and STIs prevention are needed. Teenage pregnancy rates in Kenya had remained high for two decades at about 18% (9) but have recently declined to 15% (10). Persistently high teenage pregnancy leads to 10,000 girls facing stigma and dropping out of school every year thus slowing the efforts of the Government to attain a 100% transition to secondary schools and colleges. Some of the pregnant girls may become child brides with heightened risks of more unintended pregnancies and even gender-based violence (GBV) (11). Unintended pregnancies among teenagers more often than not result in poor health outcomes such as unsafe abortions, miscarriages, stillbirths, and complications during births that may result in infant and/or maternal mortality. Short inter-pregnancy intervals that pose greater risks of morbidity and mortality in mothers and newborns is another negative phenomenon (1). The use of contraception contributes to a reduction in maternal deaths because it helps delay/space births such that women get pregnant when they are ready and this reduces rates of unsafe abortions from unwanted pregnancies. It also contributes to reduced neonatal mortality rates and improved child survival while women with more children are protected from the possible negative effects of more births (12). Women aged 15–19 in Kenya contribute 31% of maternal mortality and have a much higher mortality rate at 464 deaths per 100,000 live births against a national average of 355 deaths per 100,000 live births (2). If modern contraception was fully provided for adolescent girls, unintended pregnancies would drop by 73% annually while 7 in 10 abortions would likely be averted. Additionally, if quality healthcare was accessible for all pregnant adolescents and their newborn babies, the high adolescent maternal mortality would drop by 76% from 464/100,000 to 110/100,000 (13).

The Kenya Demographic and Health Survey (KDHS) 2014 showed that most sexually active 15–19-year-olds were not using contraception and only half of those using were consistent while 14% were ambivalent about getting pregnant. Ninety percent of the unmarried sexually active adolescent girls reported wanting to avoid pregnancy in the next two years but less than 50% were using a contraceptive method. The group accounted for about 14 percent of births in Kenya and had a high unmet need for contraception at 21% against the national average of 18% (9). The contribution to unmet need by adolescent girls aged 15–19 was high comprising 86 percent of unintended pregnancies in Kenya. Among adolescent girls, 2 in every 3 pregnancies were unintended while 35% of the pregnancies resulted in abortion. Unmarried, sexually active adolescent girls, had 44% unmet need for modern contraception as compared to 32% for married adolescent girls (14). The challenge of unmet need to the success of family planning programs is aptly analogized to the leaking bucket phenomenon in family planning where discontinuing users continually trickle into a bucket of past and never-users. This trickle continually increases the bucket of unmet need and never allows the bucket of contraceptive users to rise substantially (15).

Method mix is vital in contraceptive use dynamics because it gives women more opportunity to choose the method that works best for them hence it influences trends in fertility. The use of more effective methods of contraception has been associated with greater fertility declines than the use of less effective methods. A limited method mix does not encourage switching because a woman/couple may face the challenge of side effects or inconvenience from the method they are using but lack a more suitable one to switch to and hence discontinue. This may raise discontinuation rates and hence unintended pregnancies (16).

KDHS 2014 data highlighted the sizeable share of the modern method mix in Kenya that the 15–24 age group accounted for among all users of reproductive age. The shares were; 24% injectable users, 18% implants users, 15% pill users, and 8% of IUD users. It also revealed the shifting popularity from injectable to implants for young women but episodic and incorrect use remained a challenge and unintended pregnancies prevailed among the group (9). However, provider bias and peer influence may likely influence youth to use short-acting contraception (SAC) such as pills, condoms, and injectables which are associated with higher discontinuation rates because they are highly dependent on user adherence. Long-acting reversible contraception (LARC) is 99% effective if used correctly but may not be accessible to youth in spite of the policies recommending them for even unmarried and nulliparous (without children) youth. They may also have non-contraceptive benefits like reducing menstrual flow and pain hence reducing endometriosis (17).

Age directly impacts contraceptive use with younger women mostly using short-term methods to prevent or delay pregnancy. Women aged 15–24 are mostly typical users with lower compliance and consequently higher rates of unintended pregnancies. Studies have shown that more than 90% of sexually active young women get pregnant within a year of not using contraception (3). The main reasons for adolescents not using contraceptive services are concerns about confidentiality and side effects. In Kenya, adolescents below 19 years constitute 20% of women getting post-abortion care treatment in health facilities and 50% of subsequent admissions with severe complications (18). The urgent need to expand access to safe and affordable reproductive health care and options for vulnerable young women who procure unsafe abortions cannot, therefore, be overstated. Evidence has found that the use of contraception is a primary factor in reducing adolescent fertility. Enhanced contraceptive information, counseling and services, and the use of effective contraception have enabled many young women to avoid pregnancy and go on to attain their educational and economic goals (19).

Investing in family planning is highly cost-effective and may reduce the costs of healthcare and cascade benefits to a big section of the population. Kenya cumulatively saved KSh381 ($4.48) per every KSh85 (US$1) spent on family planning in direct healthcare costs in 2015 (20). Additionally, Kenya's demographic window is expected to open in 2038 and strategic investments in family planning are one of the proposed accelerators towards the achievement of the Demographic Dividend (DD) which is expected to be realized by 2050. The DD is projected as a five-fold economic growth that will address most economic challenges faced by Kenya. To achieve a demographic dividend, Kenya must undergo a demographic transition where lower birth and death rates prevail because of the strategic investments in family planning. Contraceptive use is a game-changer in reducing fertility and the uptake momentum that may be generated by high contraceptive use among young women aged 15–24 could result in the requisite fertility declines towards a demographic transition. The annual rate of population growth could also be slowed if the women delayed their first births and Kenya may thus be able to achieve the 2030 target of 2% growth rate (21).

The use of contraception also enhances gender equality and women empowerment as it allows women to attain higher education hence an improved social status where they are able to decide on the timing, number, and spacing of their children. The use of contraception also enables the quantity-quality trade-off where couples are able to provide a higher quality life for the fewer children they have with less financial constraints than if they were providing for more children (22). Kenya had remarkable progress in the uptake of contraception and attained a contraceptive prevalence rate (CPR) of 58% for married women in 2014 and a total fertility rate (TFR) of 3.9 and the focus then shifted to expanding equitable access in the counties. KDHS 2022 has revealed more progress with a CPR of 63% for married women for all methods and 57% for modern methods and in line with the increasing CPR, TFR has declined to 3.4 children. Use of any method of contraception for all women is 70%, while for modern methods is 59% (10).

In an effort to ensure every pregnancy is wanted, Kenya has been commemorating World Contraception Day (WCD) every year since 2007 to promote access to information and encourage contraceptive methods that are safe and suitable for users. Young people under 25 years have recently been the focus with the aim of giving correct information on contraception among them and equipping them to make informed choices regarding their sexual and reproductive health to prevent unintended pregnancies. One of the major barriers to the uptake of contraception by the cohort is myths and misconceptions about modern contraception. World Contraceptive Day 2022 sought to address this among other challenges hindering the uptake of contraception by sexually active youth. Another WCD area of focus is improving access to quality family planning services through strengthening the supply chain system and the use of technology to ensure its efficiency and effectiveness (23).

This study uses the data in the Choices and Challenges tool to show trends in contraceptive use among the 15–24 women age group with a view to providing updated data needed to form an evidence base for the high-impact policy and program efforts towards transformative reproductive health programs for the group.

## Methodology and data sources

This study examined contraceptive use among women aged 15–24 in Kenya between 2012 and 2014 using KDHS 2014 data. The KDHS 2014 data included a multiyear, month-by-month contraceptive calendar that captured individual women’s journey of reproductive decisions and documented key events, such as pregnancies, births, and contraceptive use, for up to six years preceding the survey. The calendar is therefore a retrospective contraceptive use journey from 2014 all the way back to 2009. The study is based on the contraceptive use calendar data of the 15–24 aged cohort during the years 2012, 2013, and 2014. Its visualization is derived from the Choices and Challenges tool developed by the Population Reference Bureau (PRB). PRB has analyzed Demographic and Health Survey (DHS) data from various countries and visualized it innovatively using Sankey Diagrams (24). The interactive Sankey diagrams presented show contraceptive use and continuation, non-use, switching/discontinuation, and pregnancy over a three-year period.

Sankey diagrams are an innovative visualization technique that displays the direction and flows between several variables. The categories of variables connected are referred to as nodes and the connections as links. In contraceptive use dynamics, they are used to display the often complex and changing contraceptive trajectories of cohorts of women over a period in a user-friendly format. The Sankey diagrams used in this study visualize trends in contraceptive discontinuation and switching over the period, 2012–2014. A node represents each category of family planning users or non-users. The nodes appearing at the top show the more effective family planning categories while those appearing at the bottom represent the less effective family planning categories or non-users. The links (connections) between each node are named flow lines. These flow lines display the proportion of women who started and ended in each category during the period 2012–2014. A bigger flow line implies a larger number of women flowed (moved) from one node (category) to another. Discontinuation data is mostly analyzed with the episode of contraceptive use as the unit of measurement but in the Choices and Challenges Tool, the woman is the unit of measurement to ease the interpretation of the data and promote its use in policy and program decisions (25). Data was first analyzed for all women aged 15–24 years then for contraceptive users in the same cohort. The first analysis presented data in the following categories; non-users, users of Long-Acting Reversible Contraception (LARC), users of Short Acting Methods (SAM), traditional/folk method users, and pregnant women. The second stage presented data for the share of use for individual methods namely; IUD, Implants, Injectables, Pill, and Other Modern and Traditional/folk methods. Other modern methods included male and female condoms, diaphragm, foam or jelly, lactational amenorrhea, and standard days method. Contraceptive use data is presented in Sankey diagrams while data for reasons of discontinuation and wantedness of pregnancy is presented in graphs.

The choice of 2012, 2013 and 2014 data was to have enough sample size to analyze contraceptive discontinuation and method-switching. Also, Demographic and Health Survey (DHS) contraceptive calendar data is retrospective (involves a woman's recollection of past contraceptive use), but with recent data, recall bias is reduced and quality is enhanced. The reliability and quality of calendar data have been recently evaluated using longitudinal panel data from the Performance Monitoring for Action Project and found satisfactory (26).

## Results

**Figure 1:**
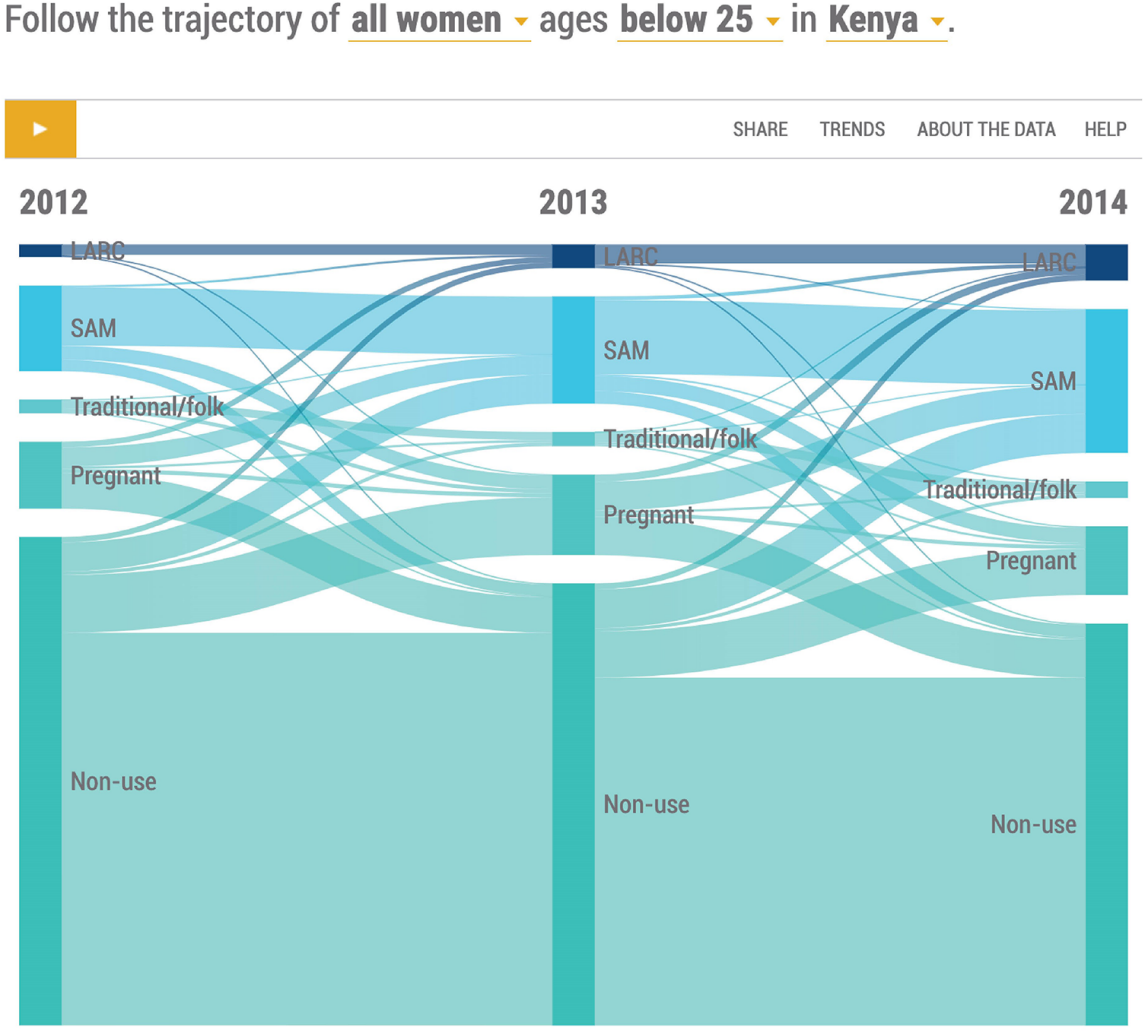
Distribution of all women age 15−24 by contraceptive use status, 2012−2014.

**Figure 2:**
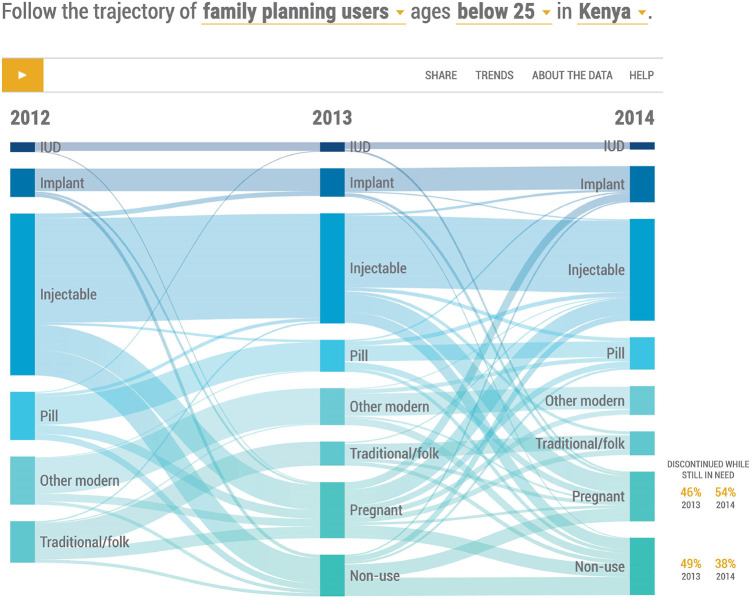
Distribution of women contraceptive users age 15−24, 2012−2014 by specific contraceptive method.

Use of contraception for women is normally dynamic and the findings presented in the following Sankey diagrams (Figures 1, 2) reflect this. Table 1 presents the actual numbers for Figures 1 and 2.

**Table 1:** Actual numbers for figures 1, 2.

Method/Status	2012	2013	2014
Figure 1—All women aged 15–24 *N* = 6,768			
LARC	128	240	367
SAM	869	1,086	1,460
Traditional/folk	140	143	168
Pregnant	677	816	697
Non use	4,952	4,483	4,076
Figure 2—Family planning users aged 15–24			
IUD	33	32	25
Implants	96	96	122
Injectable	545	371	343
Pill	162	107	109
Other modern	162	124	97
Traditional/folk	140	81	80
Pregnant	–	189	167
Non use	–	140	193
All FP users	1,137	1,469	1,995
Modern FP users	997	1,326	1,827

Derived from Choices and Challenges.

### Contraceptive use for all women aged 15–24 years, 2012–2014

Figure 1 shows all women aged 15–24 in 2012–2014 grouped into LARC, SAM, and traditional users as well as pregnant and non-users. Seventeen percent of the women were using contraception in 2012, 22% in 2013, and 29% in 2014. There was a notable increase in the uptake of contraception for both LARC and SAM and a corresponding decline in non-users. Specifically, there was a 33 percent increase in the use of modern methods in 2013 and a 38% increase in 2014. Users of traditional methods also increased by 20% between 2012 and 2014 while the share of traditional methods users reduced from 12% in 2012 to 8% in 2014.

### Non-use of FP

Figure 1 also shows an average of 66% of the women were non-users across the period. A good proportion of non-users gradually took up contraception and there was an overall decline of 18% from 2012 to 2014. About twelve percent of non-users fell pregnant in each year in 2013, and 2014. Most of the non-users who initiated contraception chose SAM especially injectable. About half of the women who were pregnant in 2012 and 2013 became non-users.

### Use of modern methods (LARC and SAM)

LARC took up a 13% share of the modern method mix in 2012, 18% in 2013, and 20% in 2014. The use of LARC was generally low but increased by 88% between 2012 and 2013 and by 53% between 2013 and 2014 although more LARC use was observed in 2014. The breakdown between IUDs and Implants is presented in Figure 2.

SAM had an 87% share of the modern method mix in 2012, 82% in 2013, and 80% in 2014. The uptake of SAM rose by 25% in 2013% and 34% in 2014 but the share of SAM in modern FP use declined between 2012 and 2014. The share of the different SAMs is also shown in Figure 2.

### Contraceptive use by method mix

Figure 2 shows the directions of outflows and inflows of users of various modern contraceptive methods as well as traditional methods from 2012 to 2014.

The injectable was clearly the most popular method throughout the period while the IUD was the least used one. About sixty-five percent of young women contraceptive users in both 2012 and 2013 continued with their current methods.

### Injectable

The injectable took the largest share of the method mix at about 50% in 2012 with sixty-four percent of young women continuing on to 2013 and into 2014. A small proportion of users experienced method failure and became pregnant each year in 2013 and 2014 while a few switched to implant and the pill. Sixteen percent and 11% of injectable users stopped altogether and became nonusers in 2013 and 2014 respectively. Eight percent of injectable users in 2014, had switched from other methods, sixteen percent had initiated the injectable after pregnancy while 6% had been nonusers.

### Implants

The share of the device in the method mix doubled in the same period from 8% to 16%. Eighty percent of users continued the method from 2012 into 2013 and from 2013 into 2014 while 11% and 3% became non-users in 2013 and 2014 respectively. The method received users who switched from the injectable and pill as well as those who initiated from nonuse.

### IUD

The IUD share of the method mix was about 4% in the period 2012–2014. Eighty-eight percent of users continued from 2012 to 2013 while 70% continued from 2013 to 2014. A few users experienced method failure.

### Pill

The pill had a 14% share of the method mix in 2012, which was reduced to 7% in 2013 and to 5% in 2014. The continuation rate was about 58% with 9% switching to IUD and injectable while 11% discontinued contraception and became non-users. In 2013, 53 percent of pill users continued into 2014 while 20 percent fell pregnant. Eighteen percent of users switched to implant and injectable while 10% became nonusers.

Overall modern method performance from 2012 to 2014 showed uptake of Implants rose by 27% while that of other methods declined as follows; IUD by 25%; Injectable by 37% and Pill by 33%.

### Other modern

Users of other modern methods took a 16% share of the modern method mix in 2012, which declined to 5% in 2014, a sixty-nine percent drop. Seventy-three percent continued the method in 2013 while fifteen percent became pregnant and about two percent switched to the pill. Only 46% of users continued with the method in 2014.

### Traditional

Users of traditional methods in 2012 reduced by 40% in 2014 hence a continuation of about 60% was shown. Thirty percent of the users became pregnant by 2014 and about 17% were lost to non-use over the period.

### Pregnancies

There was an average of 11% of pregnancies among the women across the period. The dynamics showed about 80% of the pregnancies were contributed by non-use of contraception probably because some of the women who were using contraception in 2013 may have stopped and become pregnant by 2014. SAM contributed about 75% of the pregnancies resulting from method failure with the injectable and pill being the greatest contributors. About 29% percent of the women who were pregnant in 2012 initiated SAM in 2013 while 36% of those who were pregnant in 2013 also initiated SAM in 2014.

### Discontinuation of FP

Among women who were no longer using contraceptives in 2013 as shown in Figure 2, 46 percent of pregnant women and 49 percent of non-users reported having discontinued their most recent method while wanting to avoid pregnancy. For 2014 non-users, 54 percent of pregnant women and 38 percent of non-users reported having discontinued their most recent method while still in need.

The injectable and pill had the highest discontinuations at about 30 percent including method failure while Implant had about 20 percent discontinuation. Those who switched from the injectable chose the implant, pill, and traditional methods.

### Method switching

There was some switching from one method to another, mostly from less effective methods to more effective ones in both 2012 and 2013 but it was more evident in 2013 from SAM to LARC. Switching was hardly observed in IUD and Implant methods, likely because only two years of data are examined in the diagrams but it was observed from injectable to implants and from pill to injectable and implants.

### Reasons for discontinuation of FP

**Figure 3:**
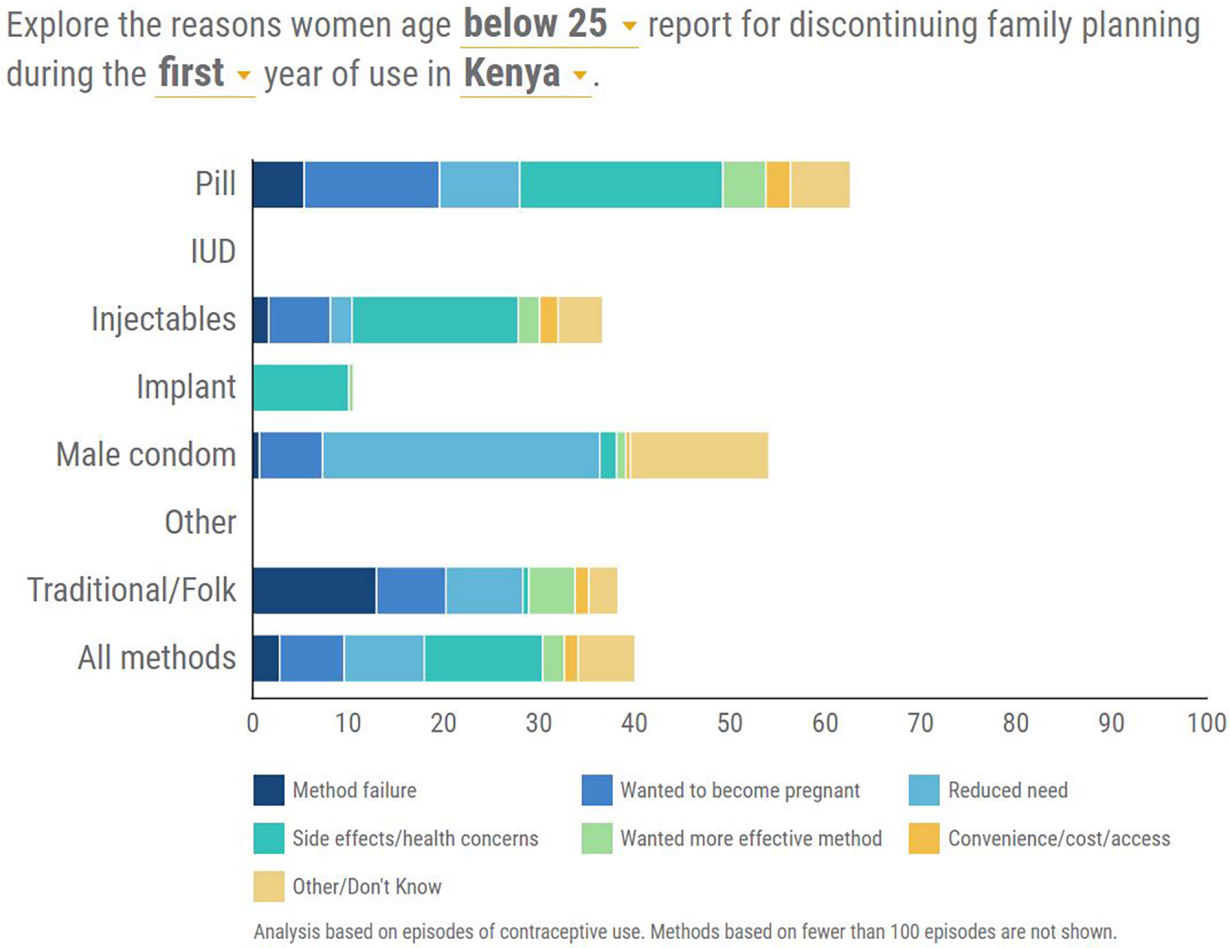
Distribution of the reasons for discontinuation of each contraceptive method between 2012 and 2013.

**Figure 4:**
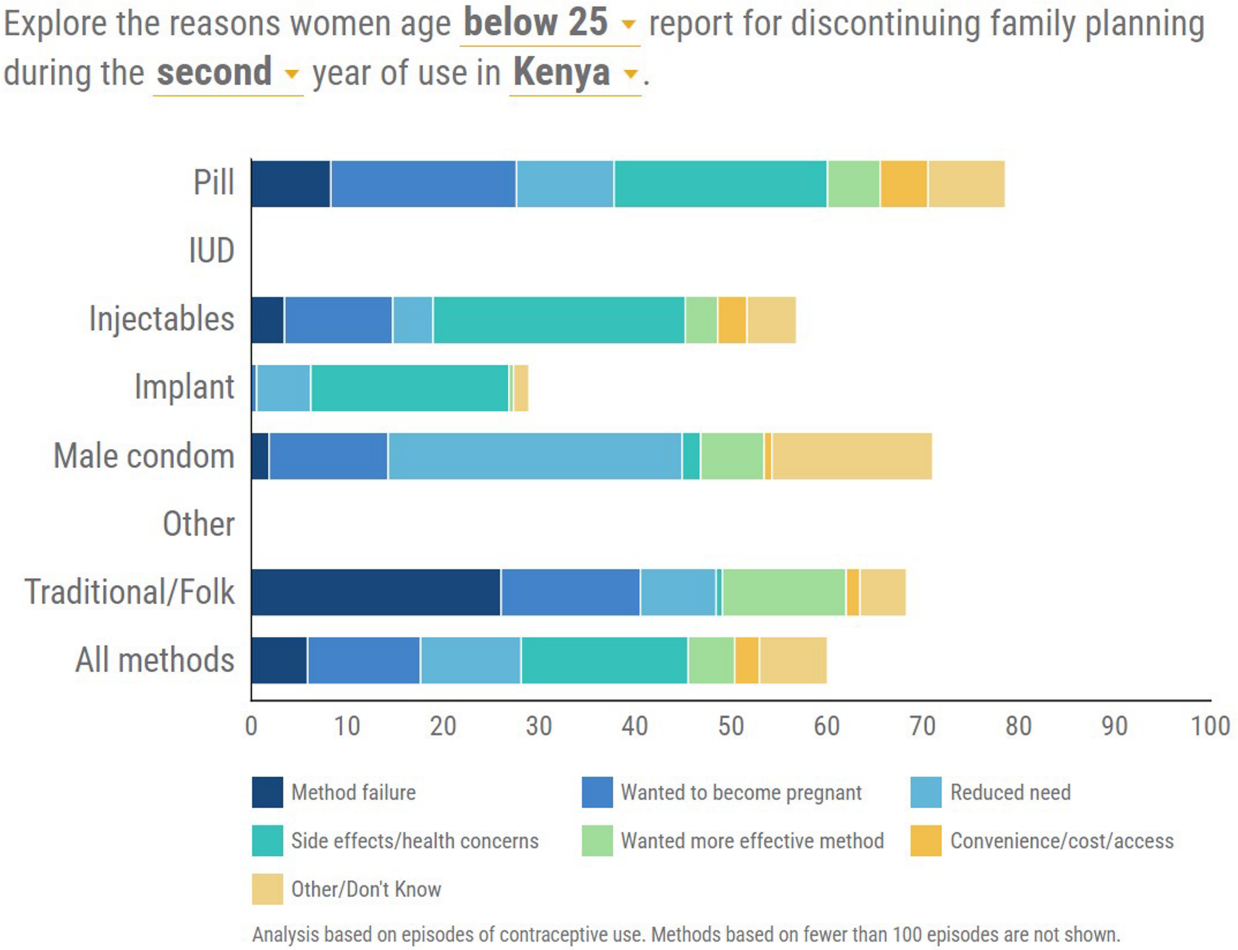
Distribution of the reasons for discontinuation of each contraceptive method between 2013 and 2014.

Of great interest were the reasons behind the decision to discontinue contraception for individual contraceptive methods. The discontinuations analyzed could have occurred any time in the period 2012–2014 and only the most recent reason for discontinuation was recorded in cases of women who had discontinued more than once. Reasons for discontinuation were categorized as shown in Figures 3, 4.

### First year of use

The leading reason for discontinuation was side effects/health concerns and this was prominent in the modern hormonal methods of Injectable, Implants, and pills in that order. Method failure was prominent in traditional methods and male condoms (Figure 3).

### Second year of use

Side effects/health concerns were more pronounced as the leading reason for discontinuation among users of hormonal methods and Injectables had the highest rates for side effects/health concerns. For the traditional method, the reason of method failure stood out while for the male condom, it was reduced need (Figure 4).

### Wantedness of pregnancy

The term “wantedness of pregnancy” is a measure of the intentionality of the pregnancy. Unwanted pregnancies may be either those that may not be wanted now or those not wanted at any future time. A wanted pregnancy is likely to lead to optimal outcomes for women and babies while an unwanted pregnancy may increase the risk of poor maternal and child health outcomes and may lead to higher rates of maternal depression and anxiety. Women with intended pregnancies have been found less likely to smoke, drink, have experienced physical abuse, or have low-birth-weight babies. Wantedness of pregnancy is not static but changes over time with changes in current circumstances and long-term goals. It is related to women's values, age, employment status, and financial and emotional circumstances among other factors.

**Figure 5:**
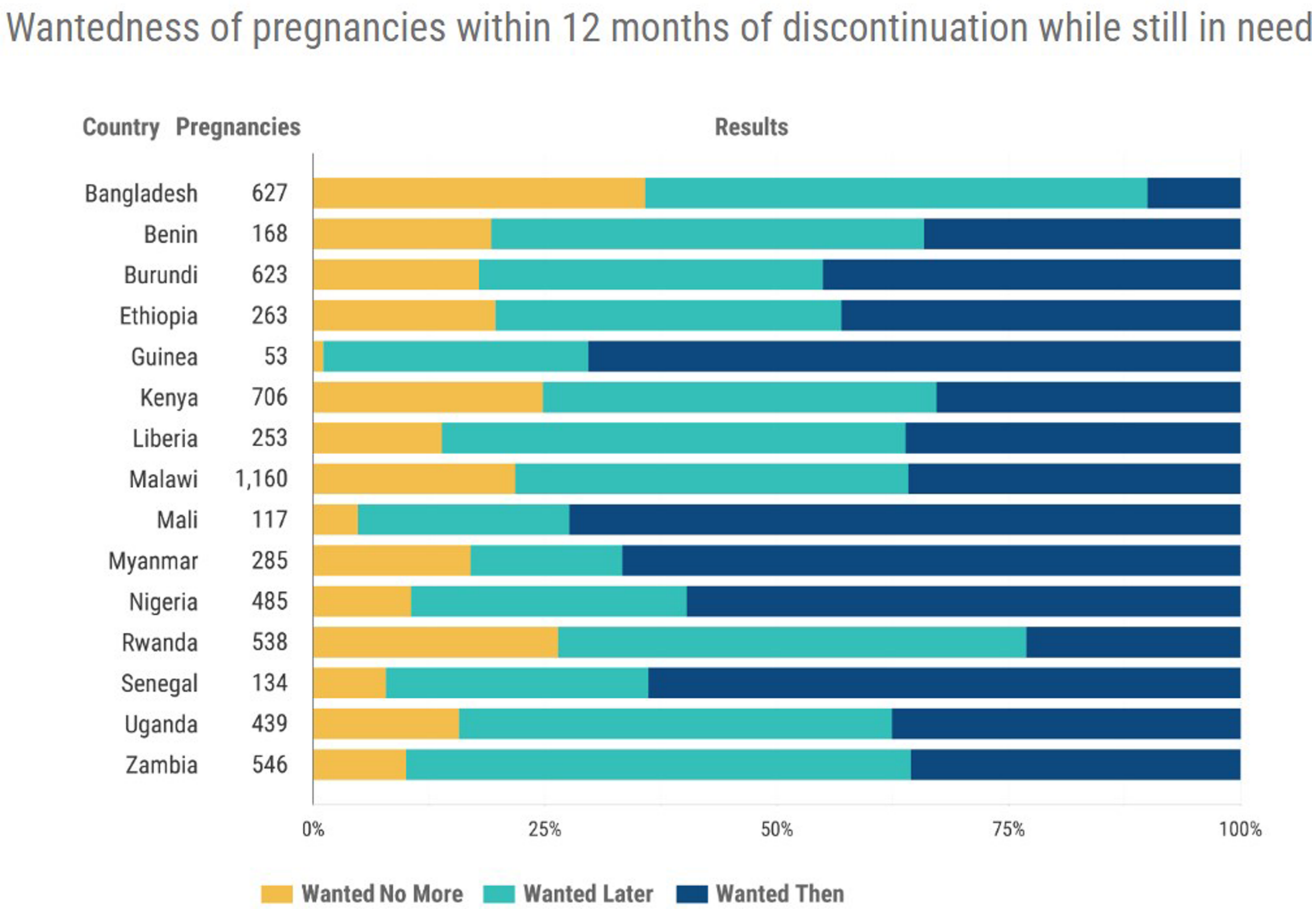
Status of wantedness of pregnancy for women, after discontinuation while in need.

Figure 5 presents a comparison of the wantedness of pregnancy data for all women in Kenya compared to 14 other developing countries mostly in Africa. The data is for women who had discontinued contraception within 12 months after initiation and while still in need of preventing pregnancy. It shows that for Kenya only about 30% of the pregnancies were wanted at the time they occurred while the unwanted pregnancies were also about 30%. It underscores the huge contribution of discontinuation to unwanted pregnancies.

## Discussion

It is important to note here the sample for the study includes all women aged 15–24 interviewed in the KDHS. Some of these women have not been involved in sexual relations and hence it could explain the low levels of use of FP. This is supported by 2014 KDHS data which shows 63% of women aged 15–19 had never had sex while 11% of those aged 20–24 also had never had sex (9). The trend has continued and 2022 KDHS data also shows 67% of women aged 15–19 had never had sex while 13% of those aged 20–24 similarly had never had sex (10). PMA Kenya 2020 data also complements the findings with 60% of 19-year-old women reporting they have never had sex while 10% of those aged 20–24 reported the same. Only 24% of women aged 15–24 reported being married and 57% of them were using modern contraception (27).

However, results from this study showed the use of contraception among women aged 15–24 rose from less than 20% to about 30% while modern method users rose by 83% in the three study years of 2012, 2013, and 2014. The use of LARC rose by 87% while that of SAM rose by 70%. Contraceptive use increases with age and marriage and therefore would increase as the women grew older and initiated sexual relations or got married. The rise in uptake of contraception is confirmed by other studies and is attributed to local and global interventions which have brought a revival of family planning campaigns that aimed to expand access for all women of reproductive age giving special focus to young women (1).

At the start of the study period, a stall in the progress of family planning indicators had been revealed by the Kenya Demographic and Health Surveys of 2003 and 2008/09 and consequently, the National Council for Population and Development (NCPD) repositioned family planning and relaunched the family planning campaigns. Additionally, NCPD developed the Population Policy for National Development (Sessional Paper No. 3 of 2012) with targets of maintaining universal knowledge of family planning and reducing teenage childbearing from 17.7 percent in 2009 to 8 percent by 2030 among others (28). Globally, the FP2020 partnership was launched at the Family Planning Summit in London in 2012 and Kenya joined the partnership which aimed to increase the uptake of modern family planning methods to an additional 120 million women and girls globally in 69 countries by 2020. Among the interventions in the Kenya commitments was the establishment of more than 70 youth empowerment centers, which would provide a “one-stop shop” for youth-friendly information, including contraception (29).

A focus area of FP2020 was the introduction of implants and IUDs as a strategy to achieve the targets of raising contraceptive uptake, lowering unmet need and expanding the method mix. The promotion of the use of LARCs in Kenya started around 2010 and more so after the development of the National Family Planning Costed Implementation Plan 2012–2016 (30) which addressed issues of supply and demand for contraceptives.

The pill was the most popular method of contraception for women age 15–24 previously but it was overtaken by injectables. Notwithstanding the effectiveness of the pill at about 99% if used correctly, there has been a shift away from its use globally towards injectables and LARCs as seen in the study results. Injectables became popular among young women because they can be used secretly and with the convenience of no daily dosage. However, LARCS give effective long-term protection and are suitable for most women including adolescents and those who have not had children (31). The increasing uptake of implants has been witnessed recently in various African countries including Kenya (32). Evidence has revealed Implants are becoming a leading method for young Kenyan women who initially chose short-term methods (33).

As recently observed, there is a general shift in Kenya from short-acting methods (SAM) towards LARCs which are more efficient, convenient, and give high user satisfaction. However, there has been more acceptability of implants than IUDs as suggested by the higher uptake of implants. This may be attributed to their simpler insertion, long efficacy, and hormonal features (34). Implants are rated to be the most effective contraceptive method and work for three to five years hence their increasing uptake among young women. The IUD is also not very popular among the cohort of 15–24 women because of its place of insertion and also because the women are mostly delaying pregnancies or spacing them.

Discontinuation of contraception among the young women in the study was 35% and side effects were the leading reason. Side effects from the mostly hormonal methods are common and knowledge on how to deal with them and the option of switching methods may be lacking in young women (4). Other contributing factors could be that a majority of the women especially in the 15–19 years category are mostly in short-term or sporadic sexual relationships, access to information on contraception and the methods is limited, and provider bias from health workers on the use of certain methods.

The high discontinuations could also be linked to low motivation to avoid pregnancy because of the ambivalence sometimes reported by young women. Qualitative studies have revealed that although women are aware of the drawbacks of childbearing, they also perceive advantages and may sometimes engage in behaviors that put them at risk of pregnancy, even when they state that they do not want to become pregnant and seek hormonal contraceptives (5, 35). User characteristics and the features of the contraceptive methods are also a major factor in method-specific discontinuation. A method like the pill may be discontinued because of its demands for high adherence and daily dosage (36). Young women may also be obtaining contraception from private sources or chemists because of convenience and higher discontinuation has been associated with non-government sources of contraception possibly because of the lack of counseling on side effects and follow-up (5, 37, 38).

The study showed about 11% of the women aged 15–24 years fell pregnant annually and for Kenya, 30% of women who discontinued contraception within 12 months while still in need fell pregnant. This is not surprising as even the United Nations Population Fund (UNFPA) has labeled unintended pregnancies as a ’silent epidemic’ with half of the pregnancies in the world being unintended (39). In another recent study, Kenya reported a rate of 46% unintended pregnancies among women age 15–19 and a much higher rate of 91% among girls aged 15–17 (40). Unintended pregnancies among adolescents mostly result in girl child school dropouts, abortions, poor maternal and child health outcomes, and poor mental health among other adverse social and health outcomes. Overall, unintended pregnancies water down the great strides in family planning and slow down the fertility transition that is expected to contribute significantly to the achievement of Demographic Dividend (DD) in Kenya by 2038 (21).

### Policy implications

Results showed low use of FP at about 30% across the period but underlying this was an increasing need for contraception outside marriage, among the study group who are mostly single but want to avoid pregnancy as they progress with education. This presents an opportunity for harnessing demand for FP and thereby raising and achieving the CPR and mCPR 2030 targets as well as accelerating the achievement of the demographic dividend and Vision 2030. This increasing demand needs a constant supply and commodity security so that adolescents and young women may prevent unintended pregnancies and abortions. Contraceptive programs should be cognizant that youth are not a homogeneous group and have varying reproductive health needs and situations that need to be addressed uniquely. Service providers should therefore give client-centered care in recognition of youth's diverse reproductive health needs.

The study showed a high prevalence of discontinuation at 35% and for women who were not using contraception including those who were pregnant, about 50% reported having had recent discontinuation. If women who discontinue do not switch to the more effective contraception, the impact on fertility may be big. Women who discontinue contraception while in need expose themselves to unprotected sex and heighten the risk of unintended pregnancies which may result in negative outcomes such as abortion, maternal and child morbidity, or even mortality. Such outcomes may roll back the good progress Kenya has attained in maternal and child health indicators. Most discontinuations were attributed to side effects and health concerns with the challenge of side effects shown to persist more in the second year of use. This raises concern about whether clients are counseled well on side effects before they initiate methods and are getting the appropriate methods. With the majority of contraceptive users being on modern methods which are mostly hormonal, the issue needs to be addressed so that women can enjoy good health and convenience while using contraception.

Data also showed almost 30% of pregnancies among women in Kenya were unintended and for the women aged 15–24 there is the thorny issue of ambivalence on pregnancy among the group. High numbers of unintended pregnancies in the population of 15–24 may have a big impact on fertility and on maternal and child health indicators. Service providers should be educated and given refresher training regularly with a focus on youth cognitive development and needs, and equipped to offer proactive, high-quality, supportive contraceptive counseling to youth. They should give non-judgmental, comprehensively clear, and concise information that addresses the facts, myths, and misconceptions about contraception as well as abstinence, dual protection, side effects, and their management. This will ensure youth make informed choices and use contraception consistently and correctly. Youth especially the unmarried may be driven by concerns of confidentiality to seek contraception from pharmacies so it is important that private providers are also empowered with training and counseling skills. Additionally, follow-up should be consistent and integrated with digital interventions.

The National Council for Population and Development (NCPD) has done a lot of work in advocacy towards ending unwanted pregnancies by improving the policy space for provision of adequate, accessible and available contraceptive information and services and commodity security with some programs especially targeted for adolescents. NCPD is coordinating a multi-sectoral campaign on ending the Triple Threat (adolescent pregnancies, new HIV infections, and Sexual and Gender-Based Violence (SGBV) across the whole country guided by action plans and technical working groups at national and county levels. Results from the Kenya Demographic and Health Survey, (KDHS) 2022 have shown some progress made with a general decline in fertility and also in teenage pregnancy from 18% in 2014 to 15% in 2022 (10). A shift towards LARC and especially implants was evident in the study but it is important to adopt a rights and choice-based approach to the provision of LARC recognizing that LARCS are provider-dependent methods and a lot of barriers in accessing and choosing them exist for the 15–24 years aged young women. LARC has been advocated as a safe option for young, unmarried, and even nulliparous women as guided by the existing policy framework and is an option for addressing the high rates of unintended pregnancies and discontinuations shown in short-acting methods (SAM) as well as the high unmet need for contraception reported for the group. The use of implants will assist young women who choose to use them to delay pregnancy for long periods and may enhance the retention of users, reduce discontinuations, and therefore unintended pregnancies among young women.

### Data quality assessment

The Choices and Challenges Tool extracts data from the most recent 2-year period of the DHS contraceptive calendar for each country. The time period chosen was to ensure the data analyzed was the most recent so as not to sacrifice data quality. Previous findings from a calendar quality assessment study undertaken by Bradley et al. (41) revealed a decline in calendar quality due to recall bias over longer time periods. Also to ensure data quality, the most recent 3 months of calendar data were excluded to account for probable pregnancies which women might have been unaware of as is the standard practice in DHS analysis. Women who reported using male or female sterilization were also excluded as they could not discontinue easily.

Limitations of the tool include the quality of the calendar data as it is retrospective; limitations in sample size when disaggregation was done by age and it could not be further disaggregated by sexual activity as it would be too small; and the use of retrospective reporting on pregnancy wantedness status.

## Conclusion

For Kenya to achieve transformative results in ending the unmet need for contraceptive and preventable maternal deaths and also achieve the targets of FP 2030, sustaining the current gains in contraceptive prevalence rate (CPR) is critical. Evidence-based approaches need to be employed to promote the retention of youth users and encourage new users so that they can attain their reproductive goals of preventing, delaying, or spacing pregnancies. High Impact Practices (HIPs) that will elevate attention and resources to assist youth in attaining their reproductive goals and avoid unwanted pregnancies should be implemented with more intensity.

## Data Availability

Publicly available datasets were analyzed in this study. This data can be found here: https://interactives.prb.org/use-dynamics/.
